# A systematic review of fully circumferential inlet patches (heterotopic gastric mucosa): More complicated than regular inlet patches

**DOI:** 10.1007/s12664-025-01738-y

**Published:** 2025-03-31

**Authors:** Daniel L. Cohen, Anton Bermont, Haim Shirin

**Affiliations:** 1 The Gonczarowski Family Institute of Gastroenterology and Liver Diseases, Shamir Medical Center, Zerifin, 7033001 Israel; 2 The Faculty of Medical and Health Sciences, Tel Aviv University, Tel Aviv, 6997801 Israel

**Keywords:** Esophageal cancer, Esophageal ulcer, Esophageal stricture, Heterotopic gastric mucosa, Inlet patch

## Abstract

**Background and Objectives:**

Inlet patches (IP) are usually small islands of ectopic gastric mucosa found in the proximal esophagus, but rare cases of large, fully circumferential IP (FCIP) have been reported. To better understand the clinical course of patients with FCIP, we sought to perform a systematic review of all published cases.

**Methods:**

A systematic review of cases of FCIP was performed according to Preferred reporting items for systematic review and meta-analysis (PRISMA) guidelines after thorough searches of PubMed and journal databases for appropriate cases. No restrictions were placed as to article type, country of origin or publication year.

**Results:**

Total 30 cases of FCIP from 27 articles were identified. These included patients from 10 different countries published between 1985 and 2024. The mean age was 55.7 with 82.1% men and a mean circumferential IP length of 3.4 cm. A majority of patients were symptomatic with dysphagia and/or a history of food impactions (72.4%). Most cases involved complications from the IP, including a benign stricture/ring/web (20, 66.7%), adenocarcinoma (4, 13.3%) or ulcers/erosions (2, 6.7%). The benign strictures were usually treated by dilation together with anti-secretory medication (10, 50%) or just dilation or medication. The adenocarcinoma cases were treated by surgery (two cases) or endoscopic resection (two cases), while the ulcer cases were treated medically. All cases with follow-up reported a good clinical outcome.

**Conclusions:**

Patients with FCIP are frequently symptomatic with dysphagia or food impactions and often have complications, including a stricture/ring or cancer. Despite this, they have good clinical outcomes. Given the risk of malignancy, endoscopic surveillance may be warranted.

**Graphical Abstract:**

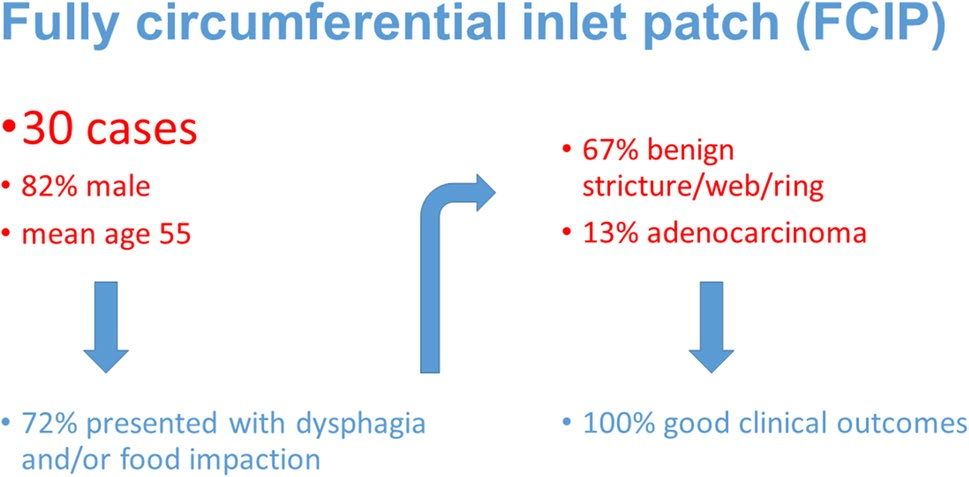

**Supplementary Information:**

The online version contains supplementary material available at 10.1007/s12664-025-01738-y.

## Introduction

Inlet patches (IP) are areas of ectopic gastric mucosa in the proximal esophagus and have several different names in medical literature. It has also been referred to as cervical inlet patch (CIP), heterotopic gastric mucosa (HGM), heterotopic gastric mucosa of the proximal esophagus (HGMPE) and heterotopic gastric mucosa of the upper esophagus (HGMUE). While initially thought to be rare, they are in fact frequently found. Recent studies, including those specifically evaluating for their presence, have found IP in 10% to 14% of patients undergoing endoscopy [1–6].

While frequently identified as an incidental lesion, there is growing data that IP can cause symptoms, many of which are attributed to their ability to produce acid [7–9]. A recent meta-analysis comparing patients with IP to those without IP identified heartburn, dysphagia, throat discomfort, globus, hoarseness and cough as being more prevalent in patients with IP [10] and endoscopic ablation of the IP has been shown to improve some of these symptoms, namely globus sensation [11–15]. Additionally, rare complications from IP have also been described such as webs, strictures, ulcer bleeding, perforation, esophagotracheal fistula and esophageal adenocarcinoma [16–19].

There is conflicting data on the correlation between the size of an IP and symptoms. Several studies have shown that larger IP size is associated with symptoms of dysphagia or chronic cough [20–22], but other studies have not confirmed this. IP, as the name implies, are usually a small round or oval “patch” or island of mucosa measuring between 2 mm and 20 mm and found in the proximal 3 cm of the esophagus [16]. However, very large IP have been described as case reports, with some of these even presenting as a fully circumferential area of gastric mucosa as opposed to just a round or oval patch. The case reports of fully circumferential IP (FCIP) suggest that these lesions may be more often associated with symptoms and complication as compared to other studies of IP.

To date, no study has attempted to systematically describe patients with FCIP. Thus, we sought to identify all of the published case reports of FCIP in literature and describe the presentation, associated findings, complications, treatment and outcomes of patients with this unique finding.

## Methods

### Search design

This study was conducted according to the Preferred reporting items for systematic review and meta-analysis (PRISMA) guidelines [23]. Searches were performed of the PubMed database. Additional searches were performed for potential abstracts presented at conferences using the databases of the American Gastroenterological Association, American College of Gastroenterology, American Society for Gastrointestinal Endoscopy and United European Gastroenterology. The search terms were as follows: inlet patch, cervical inlet patch and heterotopic gastric mucosa. Full articles, abstracts, case reports and case series, letters to the editor, images and videos were evaluated. No language or publication date restrictions were included.

### Study selection

**Fig. 1:**
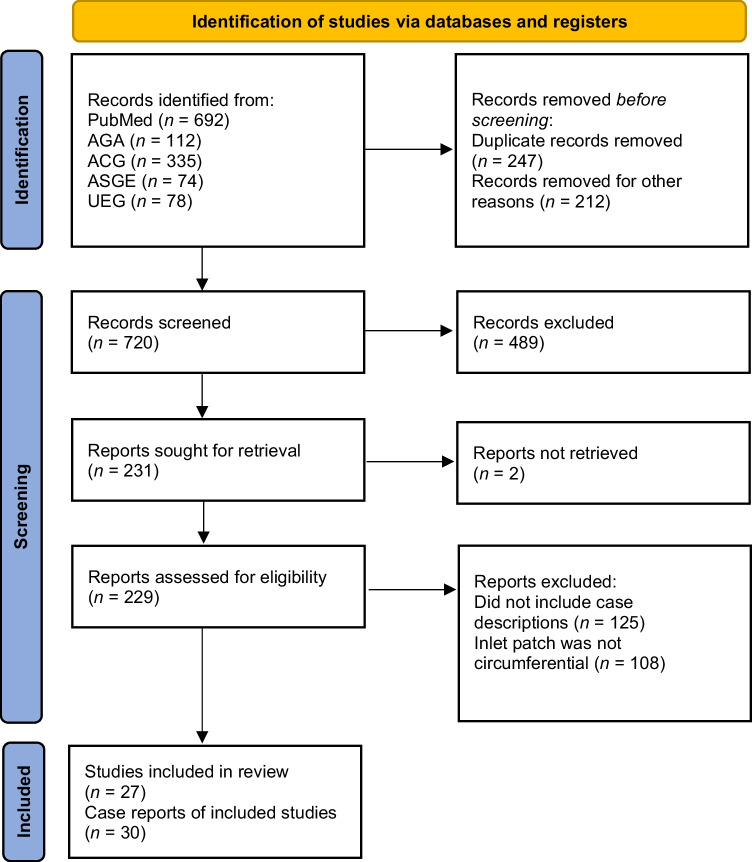
Preferred Reporting Items for Systematic reviews and Meta-Analyses (PRISMA) flow diagram of included studies.  *AGA* American Gastroenterological Association, *ACG* American College of Gastroenterology,  *ASGE* American Society for Gastrointestinal Endoscopy, *UEG* United European Gastroenterology

The overview of the literature selection process can be seen in the PRISMA flow diagram (Fig. 1). Total 1291 studies were identified from the databases according to the search terms used. After screening for duplicates and inappropriate studies, 832 studies remained. These were screened with 489 removed for not dealing with IP. After removing IP studies that did not contain descriptions of individual cases, in addition to case reports where the IP was not confirmed to be fully circumferential, 30 cases from 27 articles remained as the basis for the systematic review [24–50]. Cases were required to report that the IP was located in the proximal esophagus. Cases of nearly circumferential, but not fully circumferential, IP were excluded [51–53]. The IP length was defined as the length that the area of gastric heterotopic mucosa was fully circumferential.

### Data extraction

Data was extracted from the studies by two authors (DLC and AB). In cases of disagreement, these were discussed and adjudicated by the senior author (HS). Data retrieved from the case reports included age, gender, clinical presentation, endoscopic findings including description of the IP and its associated features or complications, histology results, treatment and patient outcome. Not all of these data points were reported or available in each case report. In those cases, the case report was still included with the available data.

### Statistical analysis

Statistical analysis was performed using Statistical Package for the Social Sciences (SPSS) version 26 for Windows (IBM SPSS Statistics, Armonk, NY. USA). Continuous variables were reported as means with standard deviations or median with interquartile range (IQR).

## Results

### Included articles

Total 30 cases of FCIP were included [24–50]. The details of the individual cases can be seen in Table 1. These 30 cases were identified in 27 articles since three articles each described two cases. The reports originated from 10 different countries and were published between 1985 and 2024. A majority were published as case reports or series, but four were published as images, three as abstracts and one as a letter to the Editor (Table 2).

**Table 1:** Details of fully circumferential inlet patch cases

Source	Year	Country	Article type	Age	Gender	Clinical Presentation	Length of FCIP	Findings associated with FCIP	Other esophageal findings	Histology of FCIP	Endoscopic treatment	Medical treatment	Surgical treatment	Patient Outcome
Jabbari et al. [24] (case 1)	1985	Canada	Research study	24	female	epigastric discomfort	3 cm	-	none	-	-	-	-	not reported
Jabbari et al. [24] (case 2)	1985	Canada	Research study	22	male	throat discomfort, nausea, vomiting	3 cm	erosions at periphery of FCIP	none	-	-	cimetidine	-	resolution of symptoms
Rattner and McKinley [25]	1986	USA	Letter to the editor	54	male	food impactions	4 cm	stricture	Barrett’s esophagus, hiatal hernia	gastric mucosa with parietal cells (fundic-type)	-	-	-	not reported
Truong ﻿et al. [26]	1986	USA	Case series	48	male	food impaction	1 cm	web/ring	distal esophagitis	gastric mucosa with parietal cells (fundic-type)	-	-	-	not reported
Powell and Luck [27]	1988	USA	Case report	5	male	dysphagia, odynophagia	1.5–2 cm	polypoid stricture	none	gastric-type mucosa	-	-	surgical resection	asymptomatic 11-years post-op
Steadman ﻿et al. [28] (case 1)	1988	Australia	Case series	63	male	dysphagia	1 cm	stricture	none	gastric mucosa with parietal cells (fundic-type)	dilation	cimetidine 400 mg twice daily	-	improvement in symptoms
Steadman ﻿et al. [28] (case 2)	1988	Australia	Case series	42	male	dysphagia	3 cm	ulcerated stricture	none	gastric mucosa (fundic-type)	dilation to 51 Fr	ranitidine 150 mg daily	-	resolution of symptoms
Buse ﻿et al. [29]	1993	USA	Case report	45	male	dysphagia	4–5 cm	web at distal end of FCIP	none	gastric mucosa	50 Fr Maloney dilation	-	-	resolution of symptoms
Bataller ﻿et al. [30]	1995	Spain	Case report	20	male	hematemesis, shock		2 ulcers (1.5 × 1.0 cm each)	none	gastric mucosa	-	omeprazole 40 mg daily	-	resolution of ulcers
Pilette ﻿et al. [31]	1995	France	Case report	67	female	dysphagia, esophageal burning	2–3 cm	ulcerated stricture at distal end of FCIP	none	gastric-type mucosa with both parietal and mucin-secreting cells	dilation	cimetidine 400 mg twice daily, followed by ranitidine 300 mg twice daily	-	improvement in symptoms
Berkelhammer ﻿et al. [32]	1997	USA	Case report	71	male	food impaction, dysphagia	7 cm	stricture within FCIP, repeat biopsies showing adenocarcinoma	small Schatzki ring	antral-type gastric mucosa, adenocarcinoma	-	-	esophagectomy	doing well with NED 2-years post-op
Pech ﻿et al. [33]	2001	Germany	Case report	77	male	nausea, vomiting	5 cm	polypoid 3 mm lesion at distal end of FCIP (adenocarcinoma)	none	gastric-type mucosa, adenocarcinoma	EMR	-	-	NED 12-months post-EMR
Rogart and Siddiqui [34]	2007	USA	Image	73	male	food impactions	-	stricture at distal end of FCIP	none	gastric-type mucosa	Savory dilation	pantoprazole	-	resolution of symptoms
Tang ﻿et al. [35]	2009	USA	Image	76	male	throat discomfort, chest pain	3 cm	-	none	gastric mucosa	-	omeprazole 40 mg twice daily	-	resolution of symptoms
Ainley [36] (case 1)	2011	UK	Case series	-	-	dysphagia	-	ring stricture at distal end of FCIP with a less severe ring at the proximal margin	none	-	balloon dilation to 16 mm	-	-	improvement in symptoms
Ainley [36] (case 2)	2011	UK	Case series	-	-	food impaction, dysphagia	3 cm	web at distal end of FCIP	none	-	balloon dilation to 15 mm	-	-	resolution of dysphagia
Afzal ﻿et al. [37]	2013	USA	Case report	75	male	dysphagia, epigastric pain	-	stricture at distal end of FCIP, erosion	none	gastric mucosa with parietal cells (fundic/body-type)	-	esomeprazole 40 mg daily	-	resolution of symptoms and healing of erosion
Lopez ﻿et al. [38]	2013	USA	Abstract	49	female	abdominal pain, nausea, vomiting	4 cm	-	none	gastric mucosa with parietal cells (fundic/body-type)	-	-	-	not reported
Blanco ﻿et al. [39]	2016	Colombia	Case report	55	female	dysphagia, globus, regurgitation, heartburn	2 cm	stricture at distal end of FCIP	none	gastric mucosa with parietal cells (fundic/body-type)	-	PPI 40 mg twice daily	-	improvement in globus
Guider and Scott [40]	2016	USA	Case report	62	male	dysphagia	3 cm	2 rings (at proximal and distal ends of FCIP)	none	cardia-type gastric mucosa	Savary dilation to 20 mm	PPI	-	improvement in symptoms
Tabibian ﻿et al. [41]	2016	USA	Case report	64	male	dysphagia, food impactions	5 cm	ring-like stricture at distal end of FCIP	none	-	balloon dilation to 15 mm	PPI 40 mg twice daily	-	resolution of symptoms
Shimamura ﻿et al.[42]	2017	Canada	Image	67	male	globus, dysphagia	3 cm	fibrotic stricture at distal end of FCIP	none	gastric mucosa with parietal cells (fundic-type)	bougie dilation to 48 Fr	pantoprazole 40 mg twice daily	-	resolution of symptoms
Yamada ﻿et al. [43]	2017	Japan	Case report	55	male	globus, dysphagia	2 cm	-	mild reflux esophagitis (Los Angeles class A)	gastric mucosa (fundic-type)	-	PPI	-	resolution of symptoms
Chang ﻿et al. [44]	2021	USA	Abstract	57	male	hematochezia, anemia	4 cm	stricture at distal end of FCIP	none	gastric mucosa with parietal cells (fundic/body-type)	-	-	-	not reported
Cohen ﻿et al. [45]	2021	Israel	Case report	68	male	food impaction, dysphagia	2 cm	stricture at distal end of FCIP	none	gastric mucosa	-	-	-	doing well
Ohki ﻿et al. [46]	2022	Japan	Case report	59	male	-	4 cm	2 cm tumor within FCIP (adenocarcinoma)	none	adenocarcinoma, slightly atrophic fundic-type gastric mucosa	ESD	-	-	not reported
Nogi ﻿et al. [47]	2023	Japan	Case report	57	male	screening	8 cm	3 cm tumor within FCIP (adenocarcinoma)	none	adenocarcinoma, gastric mucosa with parietal cells (fundic/body-type)	-	-	sub-total esophagectomy	doing well with NED 15-months post-op
Patel and Ajumobi [48]	2023	USA	Case report	78	male	dysphagia, globus	3 cm	stricture at distal end of FCIP	none	gastric mucosa with parietal cells	Savory dilation to 18 mm	PPI	-	resolution of symptoms
Pham and Coelho-Prabhu [49]	2023	USA	Abstract	76	female	dysphagia	2 cm	stricture at distal end of FCIP	none	gastric mucosa with parietal cells	dilation to 12 mm; repeat dilation to 14 mm 3 weeks later	PPI twice daily	-	improvement in symptoms
Tanaka ﻿et al. [50]	2025	Japan	Image	50	male	dysphagia, sore throat, hoarseness	5 cm	stricture at distal end of FCIP	none	gastric mucosa with parietal cells (fundic-type)	balloon dilation	vonoprazan 20 mg daily	-	improvement in symptoms

*FCIP* fully circumferential inlet patch, *USA* United States of America, *UK* United Kingdom, *PPI* proton pump inhibitor, *NED* no evidence of disease, *Fr* French, *EMR* endoscopic mucosal resection, *ESD* endoscopic sub-mucosal dissection

**Table 2:** Summary of articles included in the study

**Number of cases per article type**	
Case report	15
Case series	5
Image	4
Abstract	3
Research study	2
Letter to the Editor	1
**Number of cases per country**	
United States of America	14
Japan	4
Canada	3
Australia	2
United Kingdom	2
Colombia	1
France	1
Germany	1
Israel	1
Spain	1

### Patient demographics and presentation

**Fig. 2:**
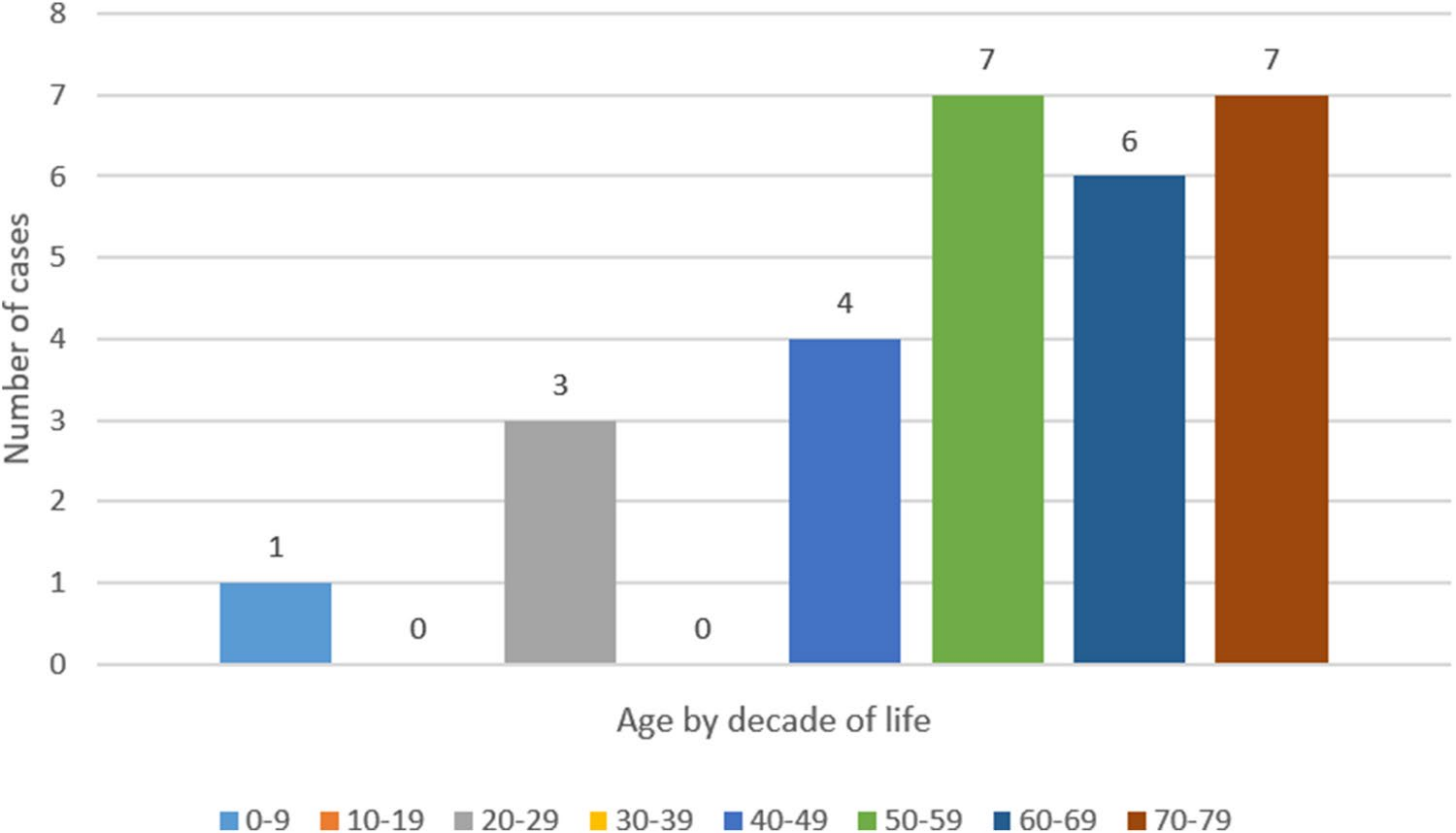
Distribution of fully circumferential inlet patch patients by decade of life

Patient demographics were available for 28 of the 30 cases (Table 3). These included 23 (82.1%) men and five (17.9%) women. The ages of patients ranged from five to 78 with a mean of 55.7 ± 18.9 (median 58.0, IQR 48.5–69.5). The distribution of ages by decade of life can be seen in Fig. 2.

**Table 3:** Summary of fully circumferential inlet patch cases

Gender (*n* = 28)	Male	23 (82.1%)
	Female	5 (17.9%)
Age (*n* = 28)	Mean ± SD	55.7 ± 18.9
	Median (IQR)	58.0 (48.5–69.5)
	< 30	4 (14.3%)
	30–59	11 (39.3%)
	> 60	13 (46.4%)
Clinical presentation (*n* = 29)	Dysphagia and/or food impaction	21 (72.4%)
	Globus	4 (13.8%)
	Nausea/Vomiting	3 (10.3%)
	Throat discomfort	3 (10.3%)
	Hematemesis	1 (3.4%)
	Asymptomatic	1 (3.4%)
Length of circumferential IP (*n* = 26)	Range	1–8 cm
	Mean ± SD	3.4 ± 1.7 cm
	Median (IQR)	3.0 (2.0–4.0) cm
Findings associated with the IP	Benign stricture/web/ring	20 (66.7%)
	Adenocarcinoma	4 (13.3%)
	Ulcers or erosions	2 (6.7%)
	None reported	4 (13.3%)
Treatment of benign strictures (*n* = 20)	Dilation plus PPI/H2B/PCAB	10 (50.0%)
	Dilation alone	3 (15.0%)
	Medication alone	2 (10.0%)
	Surgery	1 (5.0%)
	No treatment	1 (5.0%)
	Not reported	3 (15.0%)
Treatment of adenocarcinoma (*n* = 4)	Surgery	2 (50.0%)
	Endoscopic resection	2 (50.0%)
Treatment of ulcers/erosions (*n* = 2)	PPI/H2B	2 (100%)
Patient outcomes (*n* = 24)	Improvement/resolution of symptoms	24 (100%)

*PPI* proton pump inhibitor, *H2B* histamine H2 blocker, *PCAB* potassium-competitive acid blocker, * IP* inlet patches

The clinical presentation of the patient was available in 29 cases. A majority of patients presented with dysphagia and/or a history of food impactions (21, 72.4%). Globus (4, 13.8%), throat discomfort (3, 10.3%) and nausea/vomiting (3, 10.3%) were also reported.

Four patients (13.8%) appeared to have been asymptomatic from the IP. These include one patient who was diagnosed during a screening exam, one who presented with epigastric abdominal pain, one with nausea/vomiting and one with anemia. No patients had a prior history of a known inlet patch.

### Circumferential inlet patch and associated endoscopic findings

The circumferential length of the IP ranged from 1 to 8 cm with a mean of 3.4 ± 1.7 cm (median 3.0 cm with IQR 2.0–4.0 cm). There were no reports of additional IP. Biopsies from the FCIP were reported in 25 cases. All of these confirmed the presence of gastric mucosa, of which fundic-type mucosa with parietal cells was the most common histological finding (15, 60%). The other histological findings were gastric mucosa (without any further description provided) in seven cases (28%) and individual cases of combined fundic-type and antral-type mucosa, antral-type mucosa and cardia-type mucosa (4% each) (Table 1).

The FCIP was frequently associated with additional findings and complications (Table 3). Twenty cases (66.7%) reported either a benign stricture, web or ring, with a majority of these located at the distal margin of the FCIP. Three of the strictures had an ulcerated appearance or erosions. There were four cases (13.3%) of adenocarcinoma, three of which had a discrete mass-lesion within the FCIP. The other case described a stricture within the FCIP with biopsies of the stricture revealing the adenocarcinoma. Two cases (6.7%) identified ulcers or erosions without a stricture. Per the clinicopathological classification proposed by von Rahden et al. [16], 22 of the 30 FCIP cases would be considered HGM III (complicated by stricture or ulcers), with another four classified as HGM V (complicated by adenocarcinoma).

Additional esophageal findings not located in proximity to the FCIP were rare and only reported in four cases (Table 1). These included two cases of reflux esophagitis, one small Schatzki ring and one case with Barrett’s esophagus and a hiatal hernia.

### Management

Endoscopic treatment was performed in 15 cases (50.0%) (Table 1). This included 13 cases in which dilation of a stricture or ring was performed, plus two cases of endoscopic resection of an adenocarcinoma. Of the 13 cases in which dilation was performed, only one (7.7%) required a second (repeat) dilation session performed three weeks later [49], while the other 12 (92.3%) were only dilated at the initial endoscopy session. Different dilation techniques were used. Balloon dilation was performed in four cases (30.8%), Savory dilation in three cases (23.1%) and Maloney dilation in one case (7.7%), while the specific dilation technique was not mentioned in the other five reports (38.5%). No complications from endoscopic interventions were reported.

In 16 cases, anti-secretory medications were prescribed (12 cases of proton pump inhibitor (PPI), 3 cases of histamine H2 blocker (H2B), 1 case of potassium-competitive acid blocker (PCAB). Surgery was performed in three cases. This included partial esophageal resections for a benign stricture, a malignant stricture and a malignant mass lesion (Table 1).

The treatments performed varied based on the findings associated with the FCIP (Table 3). For example, those associated with a benign stricture or ring were usually treated with both dilation and anti-secretory medication (10 cases), followed by dilation alone (three cases), anti-secretory medication alone (two cases), surgery (one case) or no treatment (one case). Cases involving a malignant lesion were either treated by surgical resection (two cases) or endoscopic resection (two cases; one each of endoscopic mucosal resection and endoscopic sub-mucosal dissection). Those with isolated erosions or ulcers were only treated medically (two cases).

### Patient outcomes

Information on patient outcomes was available in 24 cases, but six cases had no information available. Remarkably, all cases with follow-up reported a good clinical outcome with either an improvement in symptoms or complete resolution of symptoms (100%). This was true not only for patients with benign disease, but also for the three (out of four) malignant cases that included information on follow-up. Those cases, which described follow-up of 12 months, 15 months and two years, all confirmed that the patient was doing well with no evidence of residual or recurrent disease. The other case of malignancy had no outcome information available [46].

### Comparison of circumferential IP to regular IP

Table 4 shows the results for patients with FCIP from this systematic review in comparison to overall data from the literature for patients with IP in terms of prevalence, demographics, symptoms and complications [1–6, 10, 17]. This demonstrates that FCIP are much less common (< 0.1% vs. 10–14%), more likely in men (82.1% vs. 57.5%), more often present with dysphagia (72.4% vs. 16.2%) and are much more likely to be complicated by strictures (66.7% vs. < 0.1%) or malignancy (13.3% vs. < 0.1%) than regular non-circumferential IP.

**Table 4:** Comparison of fully circumferential inlet patches to overall inlet patches

Variable	Fully Circumferential Inlet Patch	Overall Inlet Patch
Prevalence	< 0.1%	10–14% [1–6]
Mean age	55.7	51.0 [10]
Male gender	82.1%	57.5% [10]
Dysphagia	72.4%	16.2% [10]
Throat discomfort	10.0%	20.1% [10]
Globus	13.3%	12.7% [10]
Stricture/web/ring	66.7%	< 0.1% [17]
Malignancy	13.3%	< 0.1% [17]

## Discussion

This review shows that patients with rare finding of FCIP often present later in life, are male, have dysphagia and are complicated by a stricture/ring/web or malignancy. Despite the complications, they appear to do very well clinically with treatment.

Despite first being described by Schumidt in 1805, IP is still not clearly understood [54]. Post-mortem studies of children showed a prevalence of 4.5% [55]. More recent endoscopic studies have shown rates of 10% to 14% in patients undergoing upper endoscopy [1–6]. However, the prevalence of FCIP appears to be much lower. For example, in seven studies detailing 1295 consecutive patients with IP, not a single case of FCIP was reported [6, 56–61]. Only in a study by Cheng et al. with 99 IP patients was one FCIP noted [62]. Given these studies, the rarity of FCIP becomes clear with the prevalence of FCIP likely far less than 1% of all IP. This may explain why only 30 case reports are available in literature for this review article.

There is an ongoing debate over the pathogenesis of IP [16, 18]. Some believe that IP is a congenital lesion due to the failure of squamous epithelium to replace it during fetal development, while others posit that it is an acquired lesion formed by ruptured esophageal retention cystic glands or a metaplastic transformation due to chronic acid injury. One of the interesting findings in our review is that a majority of patients with FCIP were older (Fig. 2). The fact that most of these large FCIP only presented later in life may suggest that they were not present for most of the patients’ lives and thus are acquired lesions. However, since four patients (13.3%) did present early in life (one patient at age 5 and three in their 20’s), these may be congenital cases. Thus, our findings may support both theories of pathogenesis.

We also noted that a majority of patients with FCIP were male. The association between male gender and IP has been reported in several studies [63, 64]. Indeed, a recent meta-analysis found that men were significantly more likely to have an IP than women (OR = 1.24) [10]. The reason for this is unclear and requires further study.

Dysphagia is known to be associated with the presence of IP. Recent meta-analyses of studies comparing those with and without IP have shown that dysphagia is more common in patients with IP [10, 65]. In our study, dysphagia in FCIP patients was always associated with a lumen-narrowing lesion (stricture, web or ring). These lesions are thought to be due to stricture formation due to localized acid production, a feature of which IP is known to be capable of [7–9]. Recurrent episodes of stricturing followed by a reparative healing process has been proposed as the etiology of Plummer-Vinson syndrome (Paterson-Kelly syndrome) [16, 51]. Since IP has been shown to be associated with dysphagia [10, 65] and since dysphagia in FCIP is always associated with a proximal esophageal stricture, this suggests to us that patients with non-circumferential IP may also have an element of luminal narrowing in the proximal esophagus to explain their dysphagia complaints. While a handful of cases of stricture, web or ring have been reported in non-circumferential IP [7, 25, 66–68], we suspect that there may be more cases with subtle narrowings that are not being identified.

While a vast majority of patients with non-circumferential IP are asymptomatic from it, our review showed that most patients with FCIP were symptomatic from the complications of the IP. The FCIP patients mainly presented with dysphagia and had no other cause identified on endoscopy other than the FCIP-related stricture or mass lesion. These patients also reported that their dysphagia resolved after treatment of the FCIP-related stricture or mass lesion, again support the conclusion that the FCIP-related complication was the cause for their symptoms. Using the clinicopathological classification proposed by von Rahden et al. [16], 22 of the 30 FCIP cases presented here would be considered HGM III (complicated by stricture or ulcers), with another 4 HGM V (complicated by adenocarcinoma). The high rate of complications is likely due to the greater area of gastric mucosa in FCIP leading to more acid production and complications. Thus, this supports that idea that the larger the size of an IP, the more likely it is to cause symptoms, an idea that has been suggested in other studies. For example, Baudet et al. found that larger IP size was associated with dysphagia [20], Poyrazoglu et al. showed that the median size of IP in patients with dysphagia (16.3 mm) was greater than the median size of IP in patients without dysphagia (9.9 mm) [21] and Chong et al. found that larger IP (> 15 mm) were more likely to be associated with chronic cough [22].

Four of the 30 patients (13.3%) had esophageal adenocarcinoma. Adenocarcinoma of the proximal esophagus is rare and has been shown to be related to the presence of an IP [69–71], with only 43 cases being reported between 1950 and 2013 [17]. Whether the high rate of adenocarcinoma in this study of FCIP is related to the larger size of the IP producing more acid or the large IP providing more surface area capable of dysplastic and malignant transformation is unclear. Either way, the data from this systematic review suggests that the prevalence of malignancy in patients with FCIP is much higher than that of regular IP and thus raises the question as to whether endoscopic surveillance is warranted in this specific population as it is in other high-risk populations such as patients with Barrett’s esophagus.

Finally, we found that patients with FCIP did extremely well clinically, which is very reassuring for patients. Notably, all patients with strictures responded to either a combination of endoscopic dilation and anti-secretory medication or one or the other. There were no recalcitrant strictures that could not be easily treated. Further, more invasive techniques such as ablation of the IP via either argon plasma or radiofrequency, both of which have been used to treat symptomatic IP, were not needed [11–15]. Given these positive outcomes with endoscopic and medical therapy, we suspect that the one case of a benign stricture that required surgery on a five-year-old boy (that was published in 1988) [27] would be treated differently nowadays and that treatment with dilation and PPI would likely be successful.

Our study has several limitations. First, the case reports used in this study were written by different authors and details were missing from several cases. Further, there is an inherent bias when evaluating case reports as only certain cases are deemed worthy of publication. For example, physicians may not have published cases with poor outcomes and cases of FCIP without symptoms or complications may not have been deemed enough of a priority to publish. Therefore, cases of FCIP without a stricture or malignancy may not have been published, thus falsely raising the rates of these findings in this review. Finally, not all case reports that were reviewed clearly stated if the IP was circumferential since that may not have been the focus of the author. Thus, great care was taken to include only cases where the IP was clearly circumferential.

In conclusion, we have performed the first review of patients with FCIP, a rare presentation of IP. We have shown that these patients are frequently symptomatic, often with dysphagia or food impactions due to a stricture or ring and, thankfully, appear to have a good clinical outcome. The high rate of adenocarcinoma in this population is concerning and, thus, endoscopic surveillance may be warranted. Further studies on this unique population may unravel some of the ongoing debates regarding IP such as the pathogenesis of IP and the effect of IP size on symptoms and complications.

## Supplementary Information

Below is the link to the electronic supplementary material.

Supplementary file1 (DOCX 25 KB)

Supplementary file2 (DOCX 30 KB)

## Data Availability

The data is available from the corresponding author upon reasonable request.

## Declarations

### Disclaimer

The authors are solely responsible for the data and the contents of the paper. In no way, the Honorary Editor-in-Chief, Editorial Board Members, the Indian Society of Gastroenterology or the printer/publishers are responsible for the results/findings and content of this article.
